# Meta-analysis of the effect of expression of MYB transcription factor genes on abiotic stress

**DOI:** 10.7717/peerj.11268

**Published:** 2021-06-08

**Authors:** Zhaolan Han, Xiaowen Shang, Lingxia Shao, Ya Wang, Xujun Zhu, Wanping Fang, Yuanchun Ma

**Affiliations:** College of Horticulture, Nanjing Agricultural University, Nanjing, Jiangsu, China

**Keywords:** Meta-analysis, MYB gene, Overexpression, Abiotic stress

## Abstract

**Background:**

MYB proteins are a large group of transcription factors. The overexpression of MYB genes has been reported to improve abiotic stress tolerance in plant. However, due to the variety of plant species studied and the types of gene donors/recipients, along with different experimental conditions, it is difficult to interpret the roles of MYB in abiotic stress tolerance from published data.

**Methods:**

Using meta-analysis approach, we investigated the plant characteristics involved in cold, drought, and salt stress in MYB-overexpressing plants and analyzed the degrees of influence on plant performance by experimental variables.

**Results:**

The results show that two of the four measured plant parameters in cold-stressed plants, two of the six in drought-stressed, and four of the 13 in salt-stressed were significantly impacted by MYB overexpression by 22% or more, and the treatment medium, donor/recipient species, and donor type significantly influence the effects of MYB-overexpression on drought stress tolerance. Also, the donor/recipient species, donor type, and stress duration all significantly affected the extent of MYB-mediated salt stress tolerance. In summary, this study compiles and analyzes the data across studies to help us understand the complex interactions that dictate the efficacy of heterologous MYB expression designed for improved abiotic stress tolerance in plants.

## Introduction

In their environment, plants face biotic and abiotic stress, both of which impact their health, growth, and yield (Balfagon et al., 2020; Doerner, 2020; Parmar et al., 2017). Examples of abiotic stress include temperature extremes, drought, and salt stress. More importantly, it is expected that the effects of these stressors will be exacerbated by global climate change. While plants demonstrate striking plasticity, indicating that those in nature will adapt to these changes through natural selection, this process is too slow for agricultural crops, which require breeding or genetic engineering (Kleinman, 2018; Knott & Doudna, 2018). Therefore, a better understanding of how plants adapt and adjust to abiotic stress on a physiological level, which is driven in part by the regulation of genes, proteins, and transcription factors, is of great importance (Fang et al., 2020; Rahim et al., 2019; Tang et al., 2019). This understanding will contribute to the effort to produce agricultural crops that are better adapted to harsher environments through breeding and genetic engineering.

The MYB family of transcription factors is one of the largest found in plants (Riechmann et al., 2000). Predictive analyses for the structure-function of *MYB* genes in plants have been studied since the first member was identified in 1987 (Pazares et al., 1987). MYB proteins have been shown to be essential for a wide array of biological processes, including the regulation of primary and secondary metabolism, the cell cycle, and development (Cao et al., 2020; Nguyen et al., 2019; Wen et al., 2020; Yao et al., 2020). However, they play especially crucial roles in plants in response to various abiotic stressors (Dubos et al., 2010). Numerous studies have demonstrated the involvement of MYB transcription factors in abiotic stress responses in plants. Individually, MYBs play unique roles in response to diverse abiotic stresses by regulating the expression of stress-related genes, and this function has been verified by several gene transformation studies (Agarwal et al., 2006; Ding et al., 2009; Jung et al., 2008; Seo et al., 2009). The overexpression of many *MYB* genes was found to enhance stress tolerance in various plant species (26 articles listed in Supplemental Information 1—paper). Other studies have revealed that *MYBs* improve cold tolerance by regulating anthocyanin accumulation (Leyva et al., 1995; Lin-Wang et al., 2011; Rowan et al., 2009), mediate drought response by controlling stomatal movement (Oh et al., 2011; Xie et al., 2010), and regulate cuticular wax biosynthesis in plants (Seo et al., 2011; Seo et al., 2009). During the salt stress response, MYB TFs also play key roles in suppressing or activating related genes or pathways (Cheng et al., 2013; Cui et al., 2013; Ganesan et al., 2012), with transgenic plants exhibiting improved stress tolerance and an altered internal physiological balance.

However, it is difficult to infer the mechanisms of MYB-conferred stress responses based on a single study, as heterologous *MYB* overexpression often affects the plant physiology index under both normal and stress conditions. In addition, the response of *MYB*-overexpressing plants is affected by different parameters, such as the duration of the stress exposure, type of gene recipient and donor, and different lifestyles. Meta-analysis is a statistical synthesis of data from multiple studies (Borenstein et al., 2010), so it is more reliable and consistent than analysis based on a single study. It also quantifies the range of influence of different experimental conditions on effect sizes (Auge, Toler & Saxton, 2014; Dong et al., 2018; Ma et al., 2017a). Therefore, we set out to determine whether general conclusions can be drawn from published articles by evaluating the effects of heterologous *MYB* expression on abiotic tolerance using a meta-analysis approach. Meta-analysis was conducted to assess the effects of *MYB* overexpression on drought, salt, and cold stress responses in plants, and to analyze how moderators, such as experimental conditions and materials, influence the effects of foreign *MYB* overexpression. We aim to address the following questions: (1) What are the overall effects of heterologous *MYB* expression on drought, salt, and low-temperature responses across studies? (2) Were there any differences related to *MYB* transformation between stressed and unstressed plants? (3) Which experimental variables affected the effect of heterologous *MYB* expression? We also offer advice for future research to better understand the roles of *MYB* genes in improving stress tolerance.

## Materials and Methods

### Data collection

**Figure 1:**
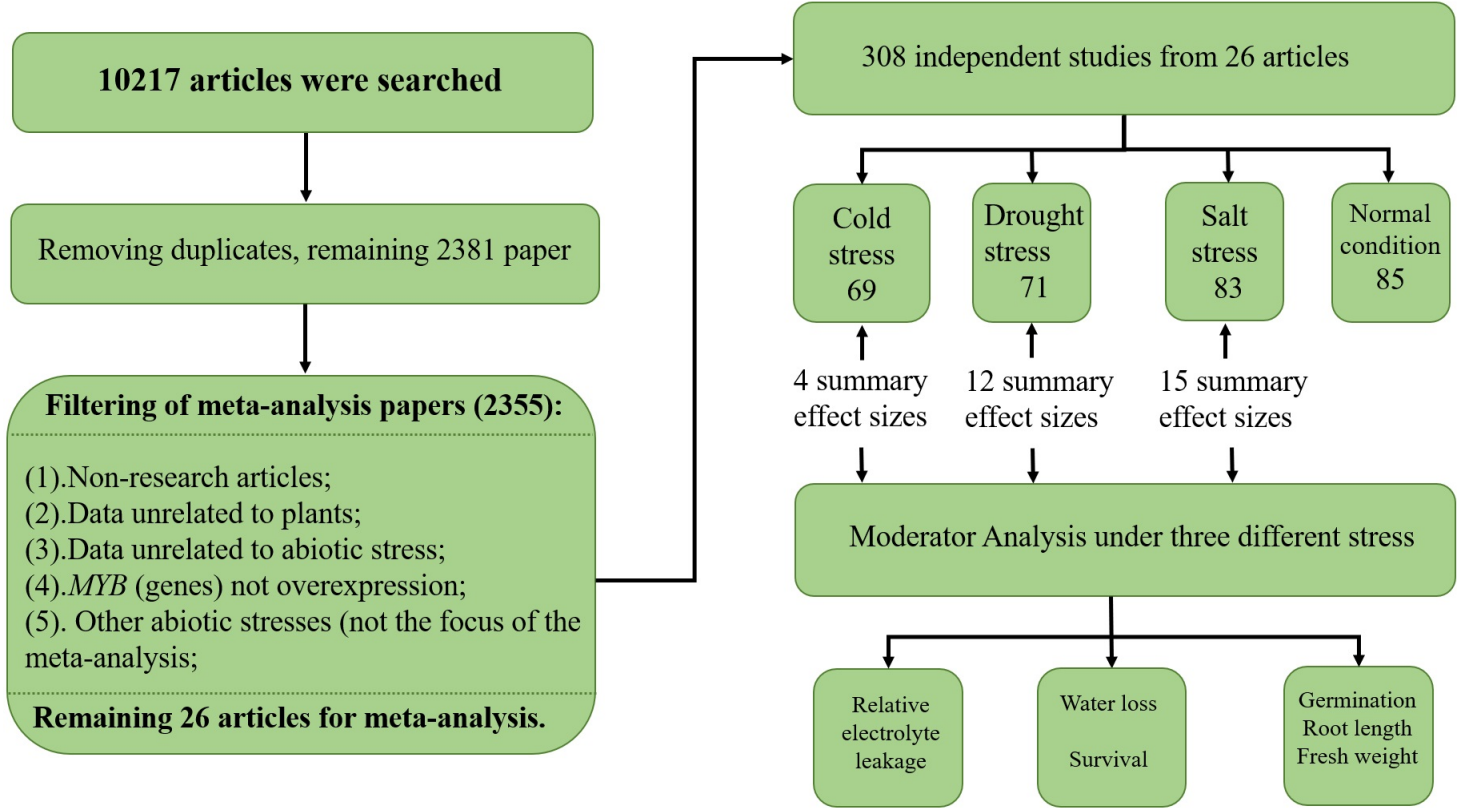
PRISMA flow diagram.

To collect studies involving *MYB* overexpression and its effect on the abiotic stress response, a systematic search of the scientific literature was conducted using six electronic databases, namely KJD, MEDLINE, BCI, SCIELO, WOS, and RSCI, with ISI Web of Science (http://apps.webofknowledge.com/) and Endnote X9 (Thomson Scientific Company). The literature search was conducted on October 9–11, 2018 using a combination of terms such as (‘*MYB* gene’/‘*MYB*’) and (‘cold’/‘salt’/‘drought’/‘overexpress’/‘transgenic’/‘stress’) (see Supplemental Information 1—search term). The initial list contained 10,217 papers, out of which 7,836 were removed as they were determined to be duplicates. The remaining papers were examined, and another 2,355 were excluded due to the following reasons: non-original research articles, 104 (comprised of 37 reviews, 57 books, nine patents, and one conference abstract); data unrelated to plants, 606; data unrelated to abiotic stress, 1,162; *MYB* gene(s) not overexpressed, 472; and the abiotic stress was not the focus of the meta-analysis, 11. In total, 26 articles, written in English, spanning 18 years were selected for the meta-analysis (see Supplemental Information 1—paper). A total of 308 independent studies were extracted from 26 papers, and if there were multiple treatments in the paper, each treatment was treated as an independent study and appeared as an individual unit in the meta-analysis—an approach commonly used for plant biology meta-analysis (Kluemper & Qaim, 2014; Lehmann & Rillig, 2015; Wujeska, Bossinger & Tausz, 2013). The breakdown of the 308 independent studies was as follows: low temperature, 69; drought stress, 71; salt stress, 83; and normal conditions, 85. The means and sample sizes of the treatments for each individual study were obtained from the original study (Fig. 1). The data from the figures in the articles were extracted using GetData Graph Digitizer v2.21 (http://getdata-graph-digitizer.com). (The authors Zhaolan Han and Yuanchun Ma performed the search strategy and data collection. When any dispute occurs, Yuanchun Ma is our manuscript referee).

### Effect size and moderators

Meta-analysis was conducted on several key response characters in cold-, drought-, and salt-stressed plants, and the data were presented as the natural logarithm of the response ratio (ln R). The response ratio in the meta-analysis results is an arbitrary unit of standardized expression, which is commonly used in the field of plant responses (Dong et al., 2018; Schaeffer, Manson & Irwin, 2013; Worchel, Giauque & Kivlin, 2013), it reflects effect size, which is often used to evaluate the overall and comprehensive effects of the study. ln R is used in the meta-analysis to indicate the log-transformed appropriate balance of positive and negative treatment effects between different response ratios (Borenstein et al., 2010; Hedges, Gurevitch & Curtis, 1999).

\begin{eqnarray*}lnR=ln{Y}_{\mathrm{TC}}{/Y}_{\mathrm{NC}} \end{eqnarray*}

where *Y*_TC_ is the mean of transgenic plants (TC) with *MYB* overexpression and *Y*_NC_ is the mean of non-transgenic plants (NC) or the mean of plants with an empty vector. ln *R*-values above 0 indicate that the TC-induced condition caused a positive effect on the parameter, whereas values below 0 indicate that the TC-induced condition caused a negative effect on the parameter, and a value of 0 indicates no effect of *MYB* overexpression on plants (Dong et al., 2018; Ma et al., 2017a; Ma et al., 2017b).

The summary effect size reflects the weighted mean of the effect sizes from the primary studies. It was computed for each plant characteristic based on at least three studies from more than one article (see Supplemental Information 1).

Moderator variables were used to determine whether the effects of *MYB* overexpression appeared to be more pronounced with specific experimental conditions compared with others. Details on the experimental design or experimental variables that might influence the response of plants to abiotic stress (low temperature, drought or salt exposure) were collected from each study. These moderators were of two types: 1) experimental conditions: treatment medium, stress severity, and stress duration; 2) experimental materials: taxonomic class (monocot or dicot), genus of the gene donor and recipient, and whether the donor and recipient were from the same genus. A graded analysis was considered if the moderator included at least two categorical levels and the data of each level was sourced from at least three studies from ≥2 articles. If the data of the categorical level did not meet the criteria, they were grouped into one level designated as “other”. This group also required at least three studies from ≥1 article. When an experiment contained more than one level of severity, we classified each level as one of four types, “low”, “chilling”, “freezing”, and “severe freezing” to evaluate whether the summary effect size was affected by different levels of stress severity. For example, when plants were subjected to cold stress, 10–25 °C was defined as “low”, 0–10 °C as “chilling”, −10–1 °C as “freezing”, and −20–11 °C as “severe freezing” (see Supplemental Information 1—moderator level).

### Meta-analysis

Comprehensive Meta-Analysis (CMA) software (v.2.2.023; Biostat, Englewood, NJ, USA; 2018) and GraphPad Prism software (v.7.00) created the forest plots. A random effect model was employed in all analyses. Some plant biology studies lacked the measurements of variation (Auge, Toler & Saxton, 2014; Dong et al., 2018; Mayerhofer, Kernaghan & Harper, 2013); therefore, the weight on the sample size (non-parametric variance) was calculated. Each primary study was weighted using nonparametric variance:

\begin{eqnarray*}V \mathrm{ln} R=({n}_{\mathrm{TC}}+{n}_{\mathrm{NC}})/({n}_{\mathrm{TC}}\ast {n}_{\mathrm{NC}}) \end{eqnarray*}

where V ln *R* reflects the variance of the natural log of the response ratio, *n*_TC_ is the number of transgenic samples, and *n*_NC_ is the number of non-transgenic samples or transgenic samples with an empty vector (Borenstein et al., 2010). The summary effect size was considered significant if *p* <  0.05, and the effect of a categorical variable was considered significant when the confidence intervals (95%) did not cross the dotted line. The sample size was used directly if it was stated explicitly in the article; otherwise, the following criteria were used to determine sample size: (1) if the sample size was not reported, *n* = 1 was used; (2) when only the LSD or standard error was given, *n* = 3 was used; and (3) if the sample size was reported as a range, the smallest value was used.

Heterogeneity, which is a measure of the real or true variation in effects, was evaluated with the Qt statistic and I^2^. The Qt statistic is based on weighted squared deviations of a descriptive index that estimates the ratio of true heterogeneity to total heterogeneity across the observed effect sizes. It is a measure of weighted squared deviations (Borenstein et al., 2010; Higgins & Thompson, 2002). The Q-test was considered significant if *p* <  0.1, which indicated significant heterogeneity in the summary effect sizes. By contrast, *I*^2^ ranges from 0 to 100%, and it reflects the proportion of true heterogeneity in the data set. The larger the *I*^2^ value, the higher the probability of heterogeneity among the studies (Iacovelli et al., 2014).

It is recognized that publication bias exists because the studies that show relatively high effect sizes are more likely to be published (Borenstein et al., 2010). Potential publication bias was examined using three methods, namely the funnel plot (Viechtbauer, 2007), Begg and Mazumdar rank (Kendall) correlation analysis (Begg & Mazumdar, 1994; Borenstein et al., 2010), and Egger’s linear regression (Harbord, Egger & Sterne, 2006). Details of these methods are provided in our previously published paper (*Ma et al., 2017a*; *Ma et al., 2017b*).

## Results

### Summary effects

**Figure 2:**
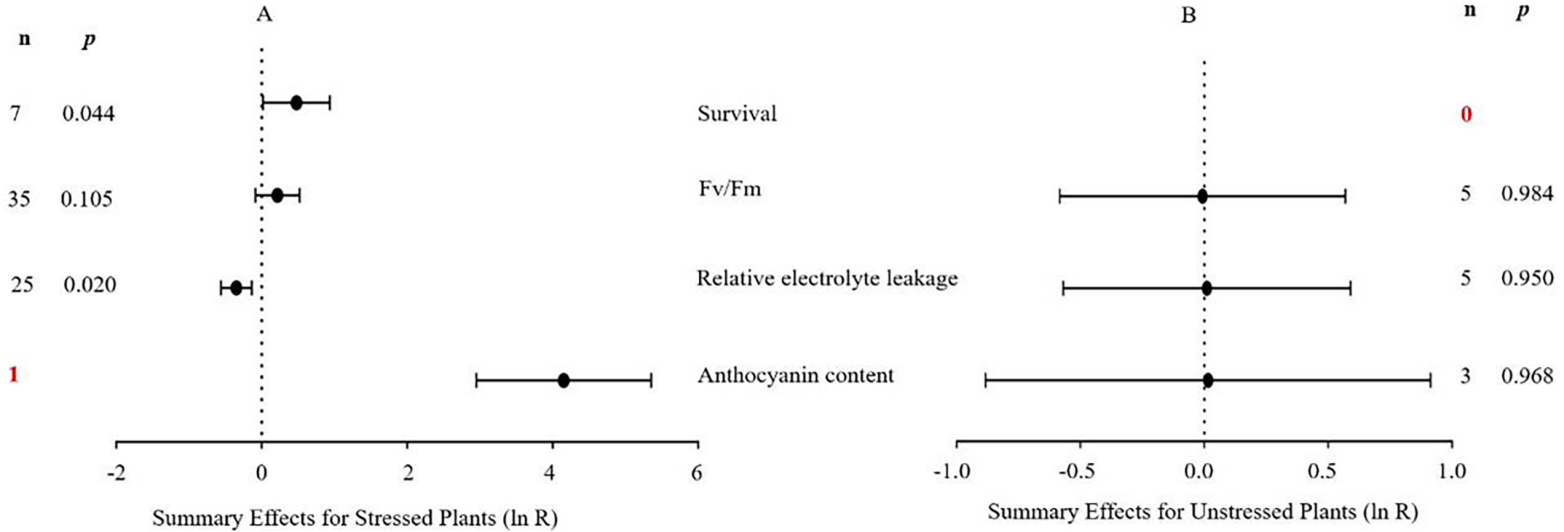
Weighted summary effect sizes (In *R*) and 95% CIs for the effect of MYB overexpression in transgenic plants subjected to cold stress (A) and non-stressed conditions (B). A *p* ≤ 0.05 indicates that the moderator level was significantly different than zero; n stands for the number of studies (same for Figs. 2–9).

**Figure 3:**
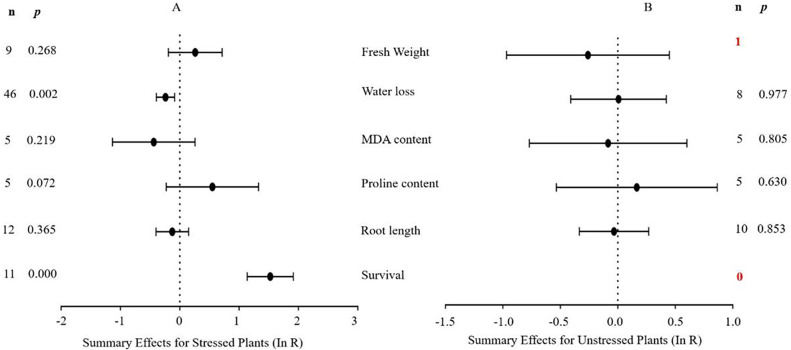
Weighted summary effect sizes (In *R*) and 95% CIs for the effect of MYB overexpression in transgenic plants subjected to drought stress (A) and non-stressed conditions (B). A *p* ≤ 0.05 indicates that the moderator level was significantly different than zero; *n* stands for the number of studies.

**Figure 4:**
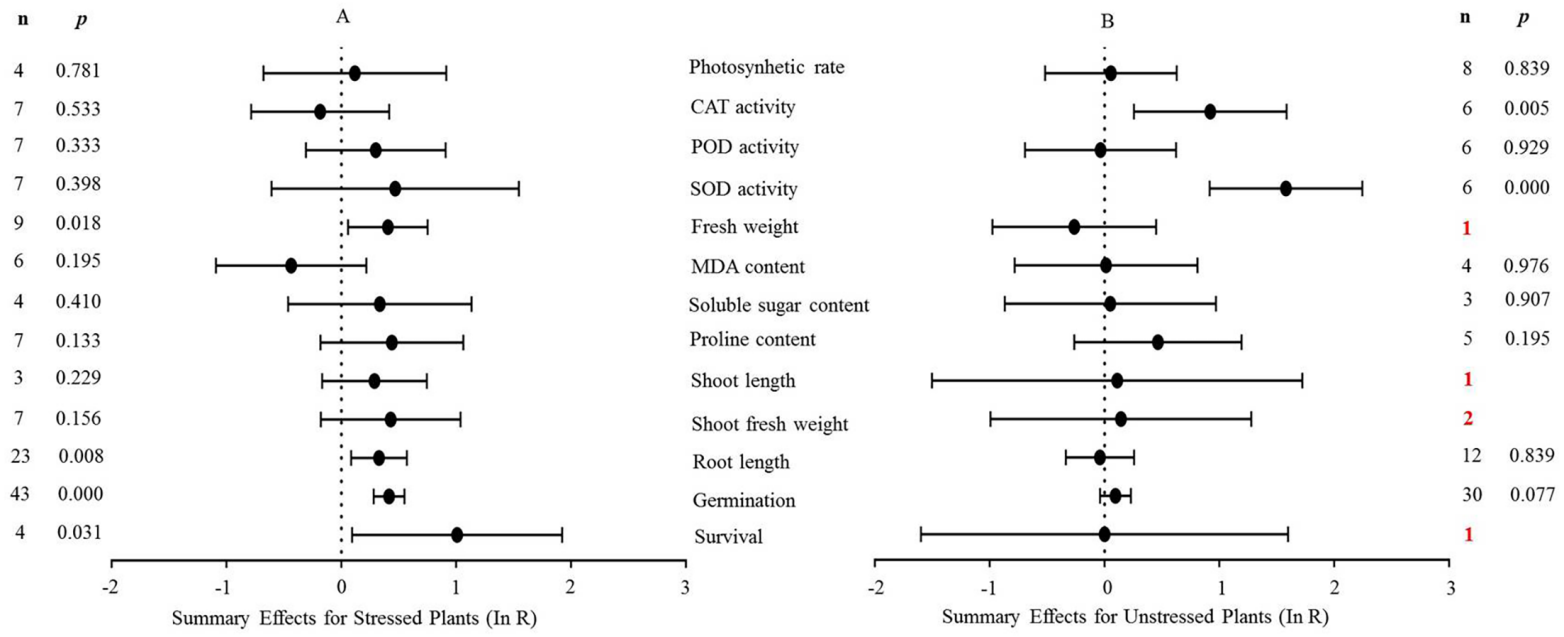
Weighted summary effect sizes (In *R*) and 95% CIs for the effect of MYB overexpression in transgenic plants subjected to NaCl stress (A) and non-stressed conditions (B). A *p* ≤ 0.05 indicates that the moderator level was significantly different than zero; *n* stands for the number of studies.

**Figure 5:**
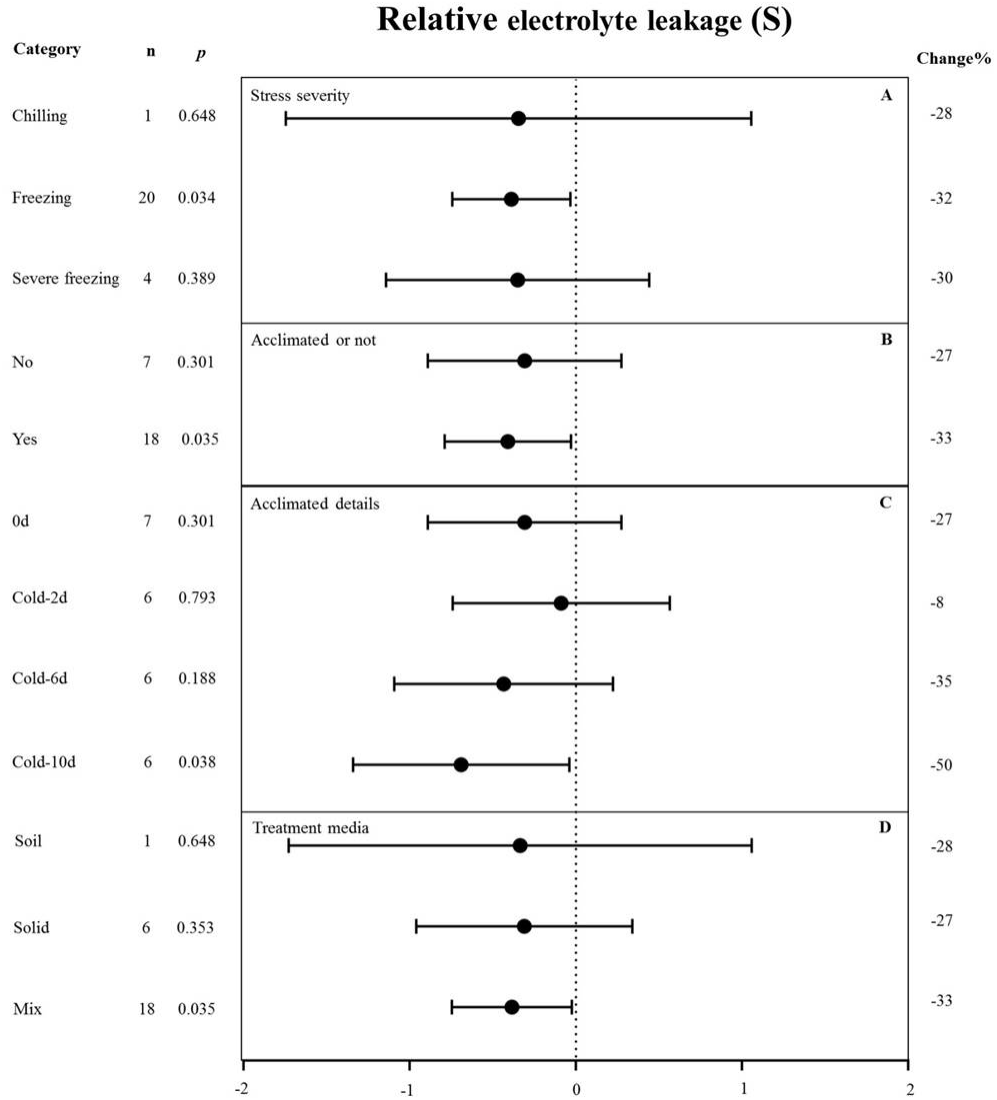
Weight summary effect sizes (In *R*) and 95% CIs showing how moderator variables affect the extent to which MYB transformation modifies plant relative electrolyte leakage under cold stress. (A) Stress severity. (B) Acclimated or not. (C) Acclimated details. (D) Treatment media. A *p* ≤ 0.05 indicates that the moderator level was significantly different than zero; *n* stands for the number of studies. Mix of treatment media represents that the solid and liquid or soil.

**Figure 6:**
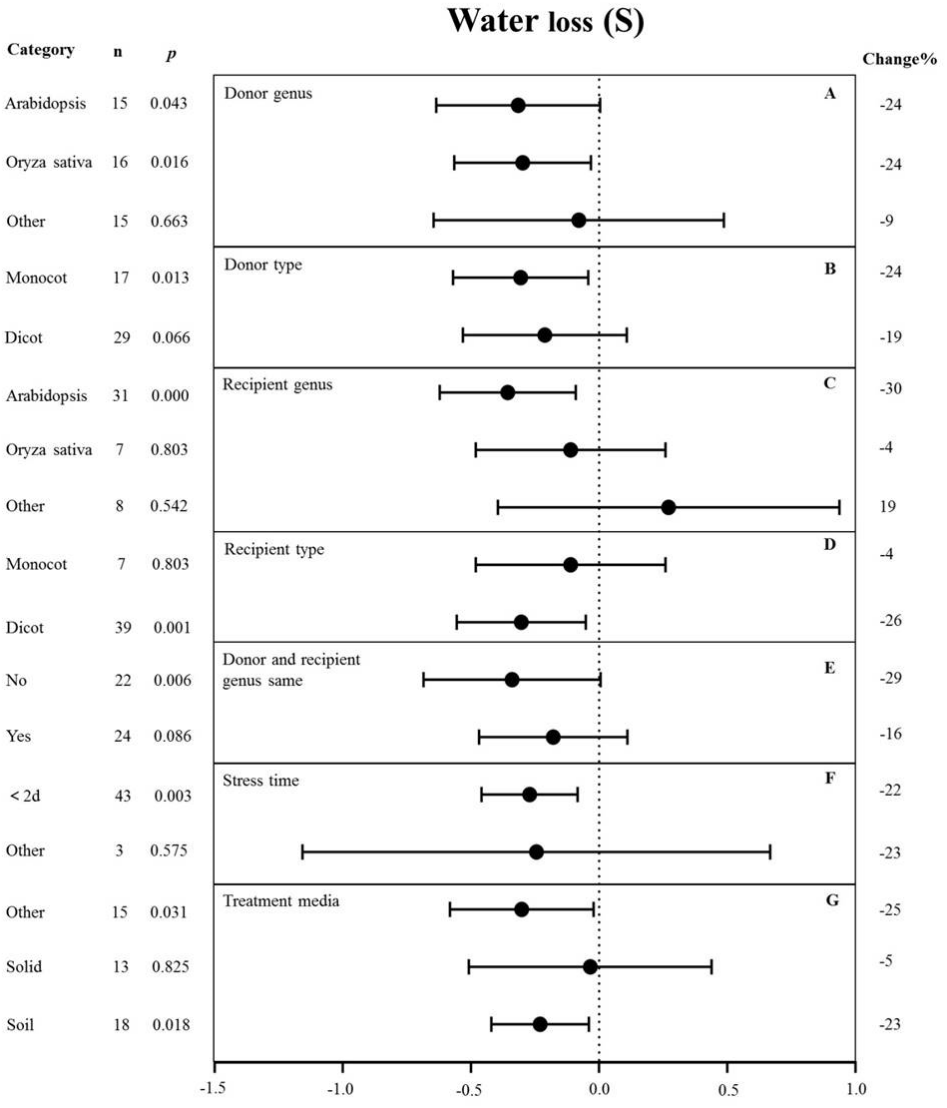
Weight summary effect sizes (In *R*) and 95% CIs showing how moderator variables affect the extent to which MYB transformation modifies plant water loss under drought stress. (A) Donor genus. (B) Donor type. (C) Recipient genus. (D) Recipient genus. (E) Donor and recipient genus same. (F) Stress time. (G) Treatment media. A *p* ≤ 0.05 indicates that the moderator level was significantly different than zero; *n* stands for the number of studies.

**Figure 7:**
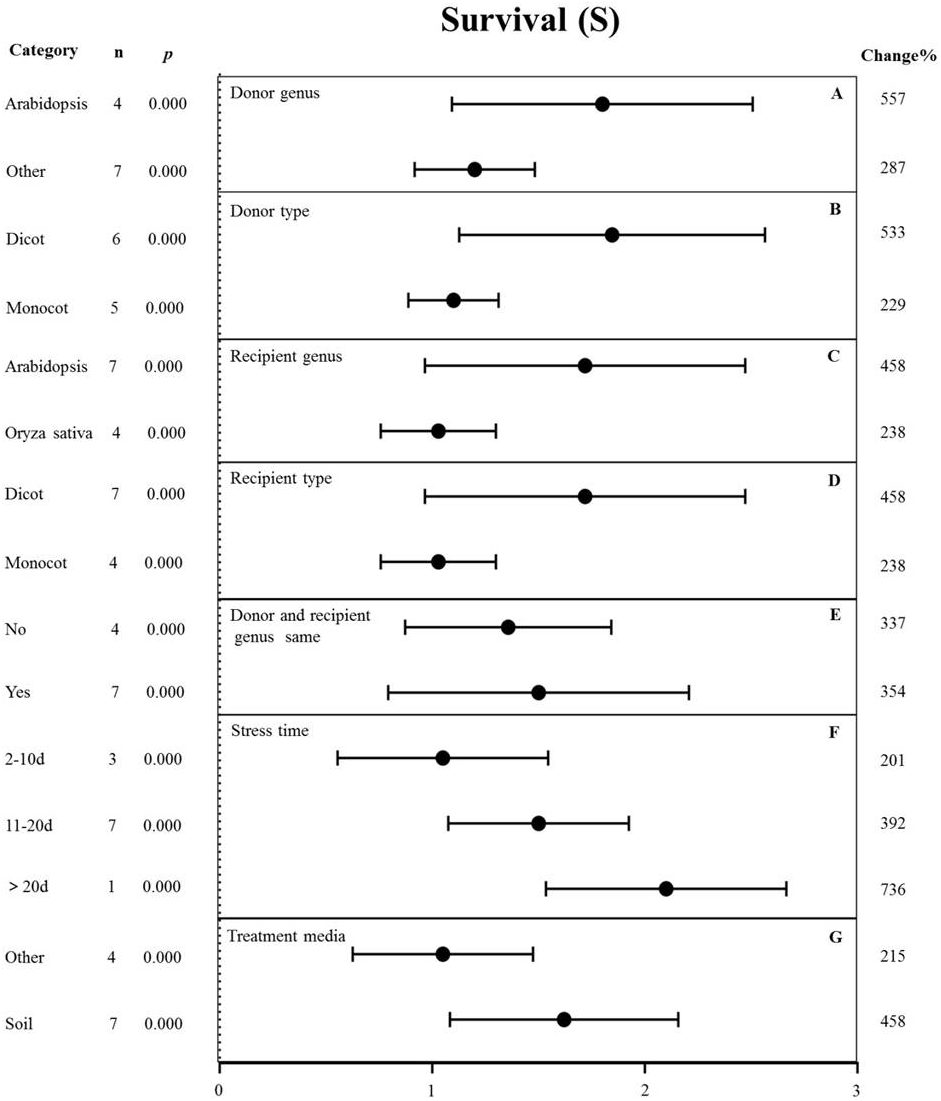
Weight summary effect sizes (In *R*) and 95% CIs showing how moderator variables affect the extent to which MYB transformation modifies plant survival under drought stress. (A) Donor genus. (B) Donor type. (C) Recipient genus. (D) Recipient type. (E) Donor and recipient genus same. (F) Stress time. (G) Treatment media. A *p* ≤ 0.05 indicates that the moderator level was significantly different than zero; *n* stands for the number of studies.

**Figure 8:**
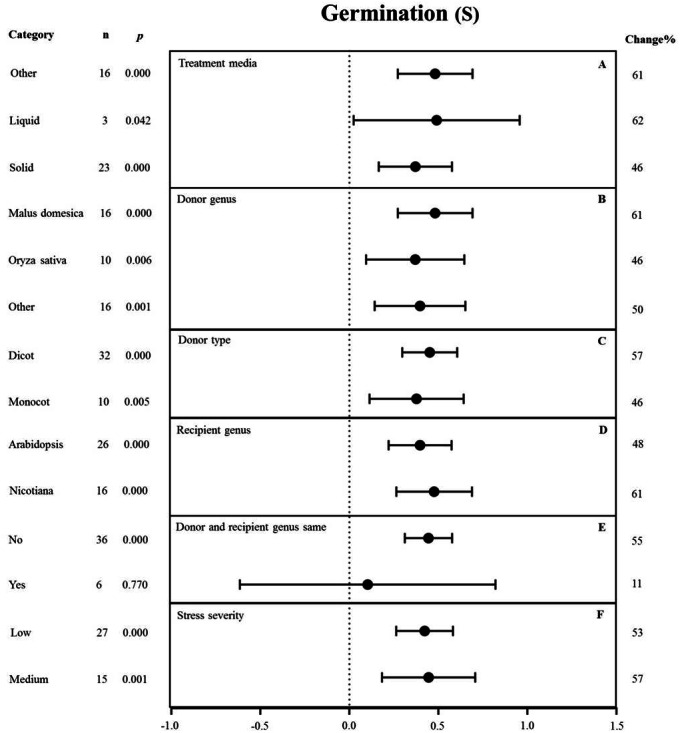
Weight summary effect sizes (In *R*) and 95% CIs showing how moderator variables affect the extent to which MYB transformation modifies seed germination under NaCl stress. (A) Treatment media. (B) Donor genus. (C) Donor type. (D) Recipient genus. (E) Donor and recipient genus same. (F) Stress severity. A *p* ≤ 0.05 indicates that the moderator level was significantly different than zero; *n* stands for the number of studies.

**Figure 9:**
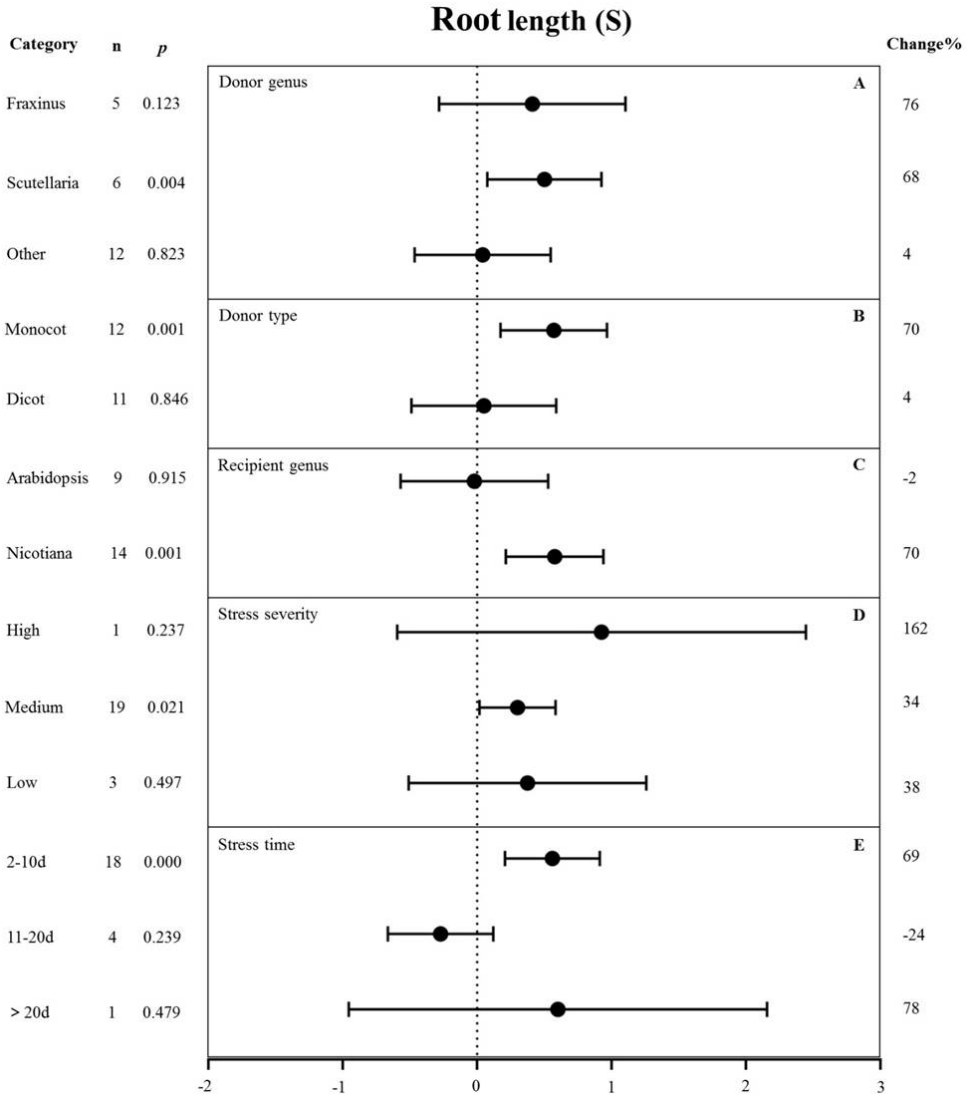
Weight summary effect sizes (In *R*) and 95% CIs showing how moderator variables affect the extent to which MYB transformation modifies root length under NaCl stress. (A) Donor genus. (B) Donor type. (C) Recipient genus. (D) Stress severity. (E) Stress time. A *p* ≤ 0.05 indicates that the moderator level was significantly different than zero; *n* stands for the number of studies.

**Figure 10:**
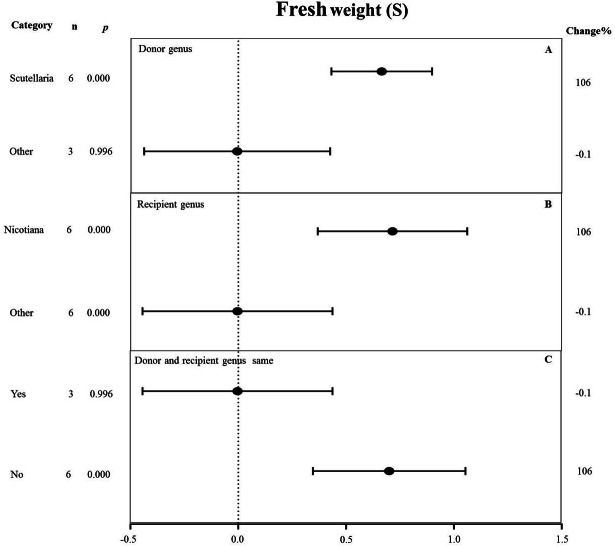
Weight summary effect sizes (In *R*) and 95% CIs showing how moderator variables affect the extent to which MYB transformation modifies root length under NaCl stress. (A) Donor genus. (B) Recipient genus. (C) Donor and recipient genus same. A *p* ≤ 0.05 indicates that the moderator level was significantly different than zero; *n* stands for the number of studies.

The natural logs of the summary effect sizes of *MYB* overexpression in transgenic plants, relative to non-transgenic plants, are shown in forest plots (Figs. 2–10), including the plants in cold, drought, and salt stress conditions, respectively, and each figure contains the effect sizes of plants under non-stress conditions. The ‘forest plot’ showed the response ratio of the transformed/non-transformed treatments and the CIs of the transformed and non-transformed treatments. ln R-values greater than 0 indicated that *MYB* overexpression had a positive effect on the effect size, and negative values indicated that *MYB* overexpression inhibited the effect size. The summary effect size reflected the relative magnitude of the effect of foreign *MYB* overexpression, and its confidence intervals (CIs) indicated its precision. The summary effects were considered significant if their 95% CIs did not overlap with 0 (*p* ≤ 0.05). The raw percentage changes induced by foreign *MYB* overexpression are listed in Tables 1–3. Based on this criterion, four summary effect sizes were computed for studies addressing cold stress, six summary effect sizes were computed for studies addressing drought stress, and 13 effect sizes were computed for studies addressing salt stress.

Four summary effect sizes in plants under cold stress and non-stress conditions are shown in Fig. 2. The results are based on five species within 82 studies (see Supplemental Information 2). The majority of the *MYB* genes were from monocots (68 studies), among which 67 were from *Oryza sativa* studies. The remaining *MYB* genes were from other studies as follows: three from *Malus domesica*, 11 from *Lycopersicum esculentum*, and one from *Triticum aestivum*. *Arabidopsis thaliana* was the most represented recipient species (68 of the 82 studies). Under cold stress, two of the four measured plant parameters were significantly impacted by foreign *MYB* overexpression (Fig. 2A, *p* ≤ 0.05, Table 1). Specifically, the summary effects of foreign *MYB* genes on the survival rate of transgenic plants were significantly higher (75%) than those on NC plants. However, the overexpression of *MYB* genes in transgenic plants resulted in a significant reduction in relative electrolyte leakage by 32% compared with NC plants under cold stress. In contrast to cold stress, the plant parameters in non-stress conditions (Fig. 2B, *p* >0.05) were not affected by *MYB* overexpression.

Figure 3 indicates the six summary effect sizes of the TC/NC response ratio in plants under drought stress vs. non-stress conditions. Meta-analysis was performed on 12 species within the 93 studies contained in 16 publications (see Supplemental Information 3). Of the 93 studies of *MYB* overexpression, eleven different species served as gene donors. *Oryza sativa* and *Arabidopsis thaliana* were the main sources of *MYB* genes (27 and 25 studies, respectively), and dicots were the most common type of donor (50 studies). In terms of recipient plants, four species were studied, with *Arabidopsis thaliana* being the most representative (51 out of 93 studies). Dicots were more common as receptors than monocots (76 out of 93 studies). Only two of the six plant parameters were significantly affected in transgenic plants overexpressing *MYB* genes when subjected to drought stress (Fig. 3A, *p* ≤ 0.05). No significant difference in the summary effect size was detected under non-stress conditions (Fig. 3B). The decrease in water loss in drought-stressed TC plants at -22% was the most prominent among the other parameters compared to NC plants at 1% after *MYB* overexpression. In addition, the survival rate of TC plants increased dramatically by 3.6-fold compared with NC plants (Table 2).

Figure 4 shows the thirteen summary effect sizes of the TC/NC response ratio in plants under salt stress conditions compared to non-stress conditions. The results of the meta-analysis were based on 143 studies involving 13 species (see Supplemental Information 4). Of the 143 studies, 12 different species served as *MYB* donors, with *Malus domesica* and *Scutellaria baicalensis* (each with 24 studies) supplying the *MYB* genes. Dicots accounted for most of the gene donors with 88 studies. Four recipient species were studied, with *Arabidopsis thaliana* (68 studies) and *Nicotiana tabacum* (69 studies) being the most representative. Dicots were more common than monocots as gene recipients (139 vs. four). Only four of the 13 plant parameters were significantly impacted in *MYB-* overexpressing plants under salt stress (Fig. 4A, *p* ≤ 0.05). By contrast, only two of the 13 plant parameters were significantly impacted by *MYB* overexpression in non-stressed plants (Fig. 4B). The survival rate increased the most among all of the parameters under salt stress as a result of *MYB* overexpression. The survival rate of TC plants was 174% that of NC plants. The fresh weight and superoxide dismutase (SOD) activity of TC plants increased by 51% and 60%, respectively, compared with NC plants under salt stress. In addition, the germination rate of TC plants was markedly improved by *MYB* overexpression under both salt stress (52%) and non-stress (12%) conditions compared with NC plants (Table 3).

### Heterogeneity

Heterogeneity, which represents the variation between studies, was determined by moderator analysis. This analysis evaluated whether the variation in observed treatment effect was over what was expected from the imprecision of the results within each study. *p*-_*hetero*_ for the *Q*-test < 0.1 or *I*^2^ >50% (Borenstein et al., 2010) indicated significant heterogeneity in the size of the summary effect, meaning that the influence of the moderator on summary effect size was inconsistent among studies (Iacovelli et al., 2014). In this case, the differences in summary effect size among moderator groups were examined using the random-effects model to determine *p*-_*hetero*_, which represents the heterogeneity among moderator classes.

The results suggested that none of the summary effects exhibited significant heterogeneity difference under cold, drought and salt stress.

**Table 1:** Heterogeneity statistics for the four summary effect sizes under cold stress and non-stress conditions.

**Summary effect size**	*Qt*	*P-*_*hetero*_	*I*^2^(%)	TC-induced change(%)
Survival (S)	5.928	0.431	0.000	**75**
Fv/Fm (S)	3.467	1.000	0.000	24
Relative electrolyte leakage (S)	2.329	1.000	0.000	−32
Anthocyanin content (S)	0.000		0.000	6724
Fv/Fm (N)	0.001	1.000	0.000	1
Relative electrolyte leakage (N)	0.216	0.995	0.000	2
Anthocyanin content (N)	0.087	0.957	0.000	2

**Notes.**

*Qt* total heterogeneity *p*-_*hetero*_ probability that *Qt* was due entirely to sampling error and not to variation among true effects *I*^2^ percentage of heterogeneity due to variation among true effects

changes to summary effects caused by foreign *MYB* genes, transformed from In R to raw percentages for TC-induced changes, bold text signifies change was significant (*p* ≤ 0.10), with positive values indicating TC-induced promotion and negative values TC-induced inhibition. (S), summary effects for stressed plants; (N), summary effects for non-stressed plants. (Same for Tables 2 and 3).

**Table 2:** Heterogeneity statistics for the twelve summary effect sizes under drought stress and non-stress conditions.

**Summary effect size**	*Qt*	*P*-_hetero_	*I*^2^ (%)	TC-induced change (%)
Fresh weight (S)	8.092	0.425	1.138	29
Water loss (S)	7.881	1.000	0.000	−22
MDA content (S)	0.221	0.994	0.000	−35
Proline content (S)	0.821	0.936	0.000	89
Root length (S)	10.741	0.465	0.000	−12
Survival (S)	10.246	0.419	2.396	360
Fresh weight (N)	0.000		0.000	−23
Water loss (N)	0.061	1.000	0.000	1
MDA content (N)	0.149	0.997	0.000	−8
Proline content (N)	0.131	0.998	0.000	19
Root length (N)	1.345	0.998	0.000	−3

**Notes.**

*Qt* total heterogeneity *p*-_*hetero*_ probability that *Qt* was due entirely to sampling error and not to variation among true effects *I*^2^ percentage of heterogeneity due to variation among true effects

Changes to summary effects caused by foreign *MYB* genes, transformed from In R to raw percentages for TC-induced changes, bold text signifies change was significant (*p* ≤ 0.10), with positive values indicating TC-induced promotion and negative values TC-induced inhibition. (S), summary effects for stressed plants; (N), summary effects for non-stressed plants.

**Table 3:** Heterogeneity statistics for the fifteen summary effect sizes under NaCl stress and non-stress conditions.

**Summary effect size**	*Qt*	*P-*_*hetero*_	*I*^**2**^(%)	**TC-induced change(%)**
Photosynhetic rate (S)	0.407	0.939	0.000	12
CAT activity (S)	2.347	0.885	0.000	−18
POD activity (S)	3.810	0.702	0.000	35
SOD activity (S)	6.000	0.423	0.000	**60**
Fresh weight (S)	8.180	0.416	2.202	**51**
MDA content (S)	0.296	0.998	0.000	−35
Soluble sugar content (S)	0.109	0.991	0.000	40
Proline content (S)	1.604	0.952	0.000	59
Shoot length (S)	0.006	0.997	0.000	33
Shoot fresh weight (S)	0.663	0.995	0.000	55
Root length (S)	17.849	0.715	0.000	37
Germination (S)	39.548	0.535	0.000	**52**
Survival (S)	2.086	0.555	0.000	**174**
Photosynhetic rate (N)	0.218	1.000	0.000	6
CAT activity (N)	3.363	0.644	0.000	154
POD activity (N)	3.011	0.698	0.000	−3
SOD activity (N)	2.159	0.827	0.000	392
Fresh weight (N)	0.000		0.000	−23
MDA content (N)	0.054	0.997	0.000	1
Soluble sugar content (N)	0.072	0.965	0.000	6
Proline content (N)	3.772	0.438	0.000	60
Shoot length (N)	0.000		0.000	12
Shoot fresh weight (N)	0.020	0.888	0.000	17
Root length (N)	1.331	1.000	0.000	−3
Germination (N)	26.330	0.608	0.000	**12**
Survival (N)	0.000		0.000	0

**Notes.**

*Qt* total heterogeneity *p*-_*hetero*_ probability that *Qt* was due entirely to sampling error and not to variation among true effects *I*^2^ percentage of heterogeneity due to variation among true effects

Changes to summary effects caused by foreign *MYB* genes, transformed from In R to raw percentages for TC-induced changes, bold text signifies change was significant (*p* ≤ 0.10), with positive values indicating TC-induced promotion and negative values TC-induced inhibition. (S), summary effects for stressed plants; (N), summary effects for non-stressed plants.

Caution should be taken during interpretation due to the low statistical power caused by the small sample sizes from available studies for certain parameters, or large differences among moderators, both of which can affect heterogeneity. Likewise, a *p*-_*hetero*_ value of < 0.1 was insufficient to demonstrate that the true effects were consistent with the summary effect. Therefore, a random effect model was used to conduct a moderator analysis of the different variables according to the summary effects significantly affected by foreign *MYB* expression to investigate the influence of different moderators (Figs. 5–10). This situation is often encountered in meta-analyses in plant biology (Ma et al., 2017a).

Of the ten summary effects that were significantly affected by foreign *MYB* gene expression, six were subjected to moderator analysis because the moderators of these six effects could be grouped into at least two categorical levels (Figs. 5–10). A total of 12 moderators were analyzed in cold, drought, or salt stress studies.

### Moderator analysis under cold stress

#### Relative electrolyte leakage

*MYB* overexpression can significantly reduce the relative electrolyte leakage (REL) under cold stress (Fig. 2). Figure 5 shows the effects of several moderators on the effect of *MYB* overexpression on the REL under cold stress. The summary effect size was determined by analyzing four moderators, namely stress severity, treatment medium, acclimation, and acclimation details, none of which significantly affected the effect size of *MYB* overexpression on the REL under cold stress (*p*-_*hetero*_ value > 0.1, Table 4). Among the stress-severity moderators, freezing had a significantly greater reduction effect on REL than chilling and severe freezing (Fig. 5A). Pre-acclimation of the plants prior to cold stress resulted in a greater negative effect on the REL (Fig. 5B). In the acclimation detail moderator, the summary effect size of REL in *MYB* transgenic plants was significantly reduced by cold treatment at 5 °C for 10 d as compared to 2 d and 6 d (Fig. 5C).

**Table 4:** Heterogeneity *p* values (*p*-_*hetero*_): probability that moderators (experimental variables) accounted for real differences in three treatments effects among studies.

**Moderator**	**Effect size**					
	Relative electrolyte leakage (Cold stress)	Water loss (Drought stress)	Survival (Drought stress)	Germination (NaCl stress)	Root length (NaCl stress)	Fresh weight (NaCl stress)
Stress severity	0.994	–	–	0.856	0.720	–
Acclimated or not	0.793	–	–	–	–	–
Acclimated details	0.632	–	–	–	–	–
Treatment media	0.966	0.642	0.094	0.770	–	–
Donor species	–	0.733	0.074	0.830	0.137	0.008
Donor type	–	0.688	0.017	0.655	0.053	–
Receptor species	–	0.085	0.130	0.551	0.033	0.008
Receptor type	–	0.173	0.130	–	–	–
Donor and recipient genus same	–	0.323	0.827	0.371	–	0.008
Stress time	–	0.964	0.246	–	0.014	–

**Notes.**

*p*-_*hetero*__≤0.1_ signifies that observed variation is not due solely to sampling error (expected variation). Dashed entries indicate that insufficient data were available to perform an analysis. Each moderator was examined in relation to the six summary effect sizes significantly impacted by MYB overexpression under cold/ drought/salt treatments. Appendix 1 provides associated *Q* values (between–study variation); *n* (sample size); *df* (degrees of freedom, levels within a moderator).

### Moderator analysis under drought stress

#### Water loss

The genus of the recipient is only one of the moderators significantly affected the effect size of MYB overexpression with regard to reducing water loss under drought stress (p-hetero value < 0.1, Table 4). The individual effects of several moderators on water loss in drought-stressed plants overexpressing MYBs are shown in Fig. 6. When Arabidopsis thaliana and Oryza sativa were used as donor genus, the effect of transformation on alleviating water loss had a comparable level of influence and reduced the water loss of TC plants by 24% under drought stress (Fig. 6A). Monocot as donor resulted in a relatively lower water loss (24%) than dicot as the donor (19%) (Fig. 6B). Arabidopsis thaliana as the recipient genus showed a 30% reduction of water loss under drought stress conditions (Fig. 6C). Likewise, dicots as recipients resulted in a relatively lower water loss (26%) than monocot recipients (4%) (Fig. 6D). Water loss was obviously lower when the donor and recipient were from different genera (29%) than when they were from the same genus (16%) (Fig. 6E). Exposure time to drought stress of <2 d resulted in a much higher level of water loss (−22%) compared with other duration times (Fig. 6F). In addition, soil as the treatment medium facilitated water loss (23% under drought stress conditions) (Fig. 6G).

#### Survival

The effects of several moderators on the survival rate of *MYB*-overexpressing plants under drought stress are shown in Fig. 7. The genus of the donor, donor type, and treatment medium significantly affected the survival rate under drought stress (*p*-_*hetero*_ value <0.1, Table 4). The effect of *Arabidopsis thaliana* (557%) as the donor on survival rate was two-fold higher than that in other genus (287%) (Fig. 7A). Dicots as the donor (533%) exhibited a 2.3-fold increase in the survival rate compared to monocots (229%) (Fig. 7B). *Arabidopsis thaliana* (458%) as the recipient genus showed a two-fold higher survival rate in TC plants than that in *Oryza sativa* (238%) (Fig. 7C). Likewise, the survival rate of dicot recipients (458%) was two-fold higher than that of monocot recipients (238%) (Fig. 7D). We observed no significant difference in the survival of transgenic plants when the donor and receptor were from the same genus or different genera. In both cases, *MYB* overexpression contributed to increased plant survival under drought stress conditions (Fig. 7E). The survival rate of *MYB*-overexpressing plants exposed to salt stress for <10 d (201%) was lower than that of plants exposed to drought stress for 11-20 d (392%). Fewer than three studies examined drought treatment longer than 20 d, and thus, there was no statistical power (Fig. 7F). The effect of *MYB* overexpression was affected the most by the planting medium, with soil-grown TC plants exhibiting an approximately two-fold higher survival rate (458%) than other types of medium (such as the solid and liquid medium) (215%) (Fig. 7G).

### Moderator analysis under NaCl stress

#### Germination

None of the moderators significantly affected the effect size of *MYB* overexpression with regard to germination under NaCl stress (*p*-_*hetero*_ value >  0.1, Table 4). The individual effects of several moderators on germination in salt-stressed plants overexpressing *MYBs* are presented in Fig. 8. The germination of *MYB*-overexpressing plants growing on solid medium was 46% higher than that of the NC plants (Fig. 8A). *Oryza sativa* (46%) as the donor genus resulted in a lower germination rate in TC-plants than that in *Malus domesica* (61%) and other genera (50%) (Fig. 8B). In the moderator category of the donor type, dicots and monocots, as recipients, had a similar effect on germination under NaCl stress, and both led to improved germination upon *MYB* expression (Fig. 8C). When *Arabidopsis thaliana* and *Nicotiana* were used as recipient genus, the germination rate of the transgenic plants significantly increased by 48% and 61%, respectively (Fig. 8D). In addition, little influence on germination (an 11% increase) was observed under salt stress when the donor and recipient were from the same genus. By contrast, the germination of transgenic plants was enhanced by 55% when the donor and recipient were from different genera under the same stress conditions (Fig. 8E). Lower stress severity promoted the effects of *MYB* overexpression on germination, and medium stress severity had a similar promoting effect on the germination of 53% and 57%, respectively (Fig. 8F).

#### Root length

Figure 9 shows the individual impact of several different moderators on the root length of salt-stressed transgenic pants. Donor genus and type, recipient genus, as well as stress severity and duration, all significantly affected the change in root length in plants subjected to salt stress (*p*-_*hetero*_ value < 0.1, Table 4). *Scutellaria* as the donor genus had the greatest promoting effect on root length (68%) compared with *Fraxinus* (76%) and other species (4%) (Fig. 9A). Dicot and monocot as donors showed differences in root length, with the *MYB* genes from monocots having a greater promoting effect on root length (70% increase compared with NT controls) than those from dicots (4% increase compared with NT controls) under salt stress conditions (Fig. 9B). *Nicotiana* as the recipient led to increased root length (70%) while *Arabidopsis thaliana* as the recipient resulted in decreased root length (2%) under salt stress compared with corresponding NC controls (Fig. 9C). The stress severity also influenced the root length of transgenic plants. The increase in root length was the greatest (34%) under medium-level salt stress conditions compared with other levels of stress severity (Fig. 9D). We observed the greatest increase (69%) in root length in transgenic plants exposed to salt treatment for 2-10 d compared with other treatment times (Fig. 9E).

#### Fresh weight

All moderators significantly affected the effect size of *MYB* overexpression for fresh weight under salt stress (*p*-_*hetero*_ value < 0.1, Table 4). *Scutellaria* as the donor genus showed the highest TC-induction effect of 106% on fresh weight compared with other genera (−0.1%) (Fig. 10A). *Nicotiana* as the recipient genus had the greatest increasing effect (106%) on the fresh weight of transgenic plants among all other genera under salt stress (−0.1%) (Fig. 10B). The promoting effects of *MYB* overexpression on the fresh weight was the greatest when the donor and recipient were from different genera (106%) compared to when the donor and recipient were from the same genus (−0.1%) (Fig. 10C).

## Discussion

Many stress-related genes are known to be directly and indirectly regulated by MYB TFs in response to abiotic stresses, suggesting that they may function at the hub of stress-related gene regulation for controlling biological processes that are involved in stress responses. For example, MYB TFs have been shown to regulate C-repeat Binding Factor (CBF1)/Dehydration Responsive Element Binding (DREB1) (Agarwal et al., 2006), REVEILLE1 (RVE1) (Meissner et al., 2013), and CIRCADIAN1 (CIR) (Guan et al., 2013) in response to cold stress. The results reported here are consistent with the findings of previous studies, which show that *MYB* overexpression positively contributes to low temperature, drought, and salt tolerance in plants. These results also highlighted the average degree of the effects across individual studies and revealed plant characteristics that have been affected the greatest by *MYB* overexpression. However, due to the low number of studies (*n* < 3) for some plant characteristics, only four summary effect sizes on low temperature, six summary effect sizes on drought stress, and thirteen summary effect sizes on salt stress were investigated. Many key characteristics were not included. For example, a previous study has reported the roles of MYBs in regulating flavonoid and anthocyanin biosynthesis, both of which have been implicated in improved abiotic stress tolerance (Borevitz et al., 2000; Fornale et al., 2014; Gonzalez et al., 2008; Heppel et al., 2013; Koops et al., 2011; Nakabayashi et al., 2014; Nesi et al., 2001; Pazares et al., 1987). However, whether the expression of anthocyanin- or flavonoid-related genes changed after *MYB* overexpression is not clear. There was only one study reporting reduced anthocyanin content upon *MYB* overexpression under cold stress. However, the small sample size rendered the results inconclusive. There are many similar physiological indicators related to resistance, such as antioxidant enzyme activity (Li et al., 2019; Li et al., 2020; Shingote et al., 2020), proline and MDA content (Gao et al., 2017; Shingote et al., 2020; Zhang et al., 2012) and so on, which are affected by overexpression MYB genes under low temperature stress. However, due to the insufficient sample size of the study, it was not included in the meta-analysis. It shows that the relevant physiological indicators of MYB in the process of resisting cold stress response are still relatively limited, which is not conducive to exploring the regulatory relationship between MYB and cold stress.

MYB TFs are also known to affect signal transduction and phytohormone biosynthesis, including auxin (Wang, Cao & Hao, 2014), gibberellic acid (Aya et al., 2009), methyl jasmonate (Liu et al., 2019; Zhang et al., 2018), and ABA (Chen et al., 2020; Xu et al., 2020; Yin et al., 2017). MYB TFs are also involved in the synthesis of plant cell walls, thereby mediating the growth and development of plant cells (Liao et al., 2008; Liu, Osbourn & Ma, 2015). However, the details on how *MYB* overexpression affects these hormonal pathways in response to abiotic stress remain to be elucidated. MYB TFs have been reported to mediate stomatal movement in response to drought (Oh et al., 2011; Xie et al., 2010). Unfortunately, among the 71 *MYB* overexpressing studies on drought stress, none investigated stomatal movement.

Salt stress results in osmotic stress and ion toxicity in plants via ion over accumulation (Isayenkov, 2012). MYB TFs confer enhanced salt tolerance by maintaining ion homeostasis (He et al., 2012; Kim et al., 2013). For example, MYBs can interact with the salt tolerance (STO) protein (Nagaoka & Takano, 2003) and suppress the expression of *salt overly sensitive* (*SOS*) genes (Kim et al., 2013) to modulate the Na^+^/K^+^ ratio. Unfortunately, in studies where *MYBs* were overexpressed under salt stress, the ion properties were not surveyed. The results of the meta-analysis based on 26 articles confirmed that *MYB* overexpression enhances stress tolerance in plants. Specifically, there was a significant increase in the survival of transgenic plants under low temperatures, drought, and salt stress (Figs. 2–4). Under low-temperature conditions, *MYB* overexpression had a positive effect on membrane permeability and significantly reduced the REL, which is consistent with the results of a previous study (Vannini et al., 2004). Under drought stress conditions, water loss in *MYB*-overexpressing plants was significantly lower than that in NC controls. Under salt stress, *MYB* overexpression exerted positive effects on root development. This result is in line with an earlier study showing that *MYB* expression is induced in the root apex under stress (Gruber et al., 2009).

According to the meta-analysis, ten summary effects showed significant changes (Table 4). However, four effect sizes were excluded from moderator analysis for not meeting the standards of subgroup analysis, namely, every moderator should be classified into two grades and the sample size of each grade should be more than two (Table 4; Figs. 5–10).

*MYB* overexpression presumably protects the cell membrane upon cold exposure, thus increasing cold tolerance. Notably, the effect of *MYB* overexpression on REL under cold stress was not affected by any moderator (Fig. 5) and was more pronounced after plants were pre-acclimated to a low temperature (Fig. 5B). For example, after 10 d of acclimation, the effect reached ∼50% compared with the control (Fig. 5C), suggesting that the effects of *MYB* overexpression during cold stress response were gradual and cumulative, rather than instantaneous.

Due to the high structural conservation of MYB TFs across divergent plant species, *MYB* homologs may have similar functions in response to abiotic stress (Li, Ng & Fan, 2015). However, there appears to be a functional aspect of MYB TF activity based on the monocot/dicot split. For example, in terms of the donor type, the heterologous expression of dicot *MYBs* gave a higher survival rate than monocot *MYBs* under drought stress (Fig. 7B). The recipient type showed a similar trend (Fig. 7D). These data suggest that the function of MYB TFs is specific along the monocot/dicot split. Further studies are required to reveal whether the function of MYB in response to abiotic stress is species-specific, as previous studies on *MYB* overexpression were performed on a relatively narrow range and the recipient genus was overrepresented by *Arabidopsis*, *Nicotiana*, and *Oryza sativa* (Figs. 6–10).

Although numerous studies have demonstrated the crucial roles of MYB TFs in abiotic stress tolerance, the extent in their functions and effects, as well as their mechanisms of action, remain to be elucidated. Here, we provide results to strengthen our understanding of *MYB* function in abiotic stress responses (Table 5). Future in-depth studies should be carried out to further understand the functions of *MYBs*.

**Table 5:** The summary effect sizes of significant changes under three different stresses.

**Summary effect sizes**		**Cold stress**		**Drought stress**		**NaCl stress**	
		TC	NC	TC	NC	TC	NC
Survival	*n* (Sample sizes)	7	0	11	0	4	1
	*p* (*p* ≤ 0.05)	0.044	0.000	0.000	0.000	0.031	0.000
REL	*n* (Sample sizes)	25	5				
	*p* (*p* ≤ 0.05)	0.020	0.950				
Water loss	*n* (Sample sizes)			46	8		
	*p* (*p* ≤ 0.05)			0.002	0.977		
Fresh weight	*n* (Sample sizes)					9	1
	*p* (*p* ≤ 0.05)					0.018	0
Root length	n (Sample sizes)					23	12
	*p* (*p* ≤ 0.05)					0.008	0.839
Germination	*n* (Sample sizes)					43	30
	*p* (*p* ≤ 0.05)					0	0.077

The rapid development of high-throughput genomic technology has brought biology into the era of ‘Big data’. The plant science community not only needs to establish a data-compatible parallel computing and data management infrastructure, but also needs to extract key information from massive data (Ma, Zhang & Wang, 2014). We suggest that a standardized set of information should be provided in each published article to help others to utilize the information in larger sets of data analytics.

## Conclusions

The meta-analysis data suggest that the treatment medium, donor/recipient species, and donor type significantly influence the effects of *MYB* overexpression on drought stress tolerance. Under salt stress, the donor/recipient species, donor type, and stress duration all significantly affected the extent of MYB-mediated salt stress tolerance. This study compiles and analyzes the data across studies to help us understand the complex interactions that dictate the efficacy of heterologous *MYB* expression designed for improved abiotic stress tolerance in plants.

##  Supplemental Information

10.7717/peerj.11268/supp-1 Supplemental Information 1 PRISMA checklist

10.7717/peerj.11268/supp-2 Supplemental Information 2 Raw data of all analysis results

10.7717/peerj.11268/supp-3 Supplemental Information 3 Raw data of all analysis results under cold stress

10.7717/peerj.11268/supp-4 Supplemental Information 4 Raw data of all analysis results under drought stress

10.7717/peerj.11268/supp-5 Supplemental Information 5 Raw data of all analysis results under salt stress

10.7717/peerj.11268/supp-6 Supplemental Information 6 Systematic meta-analysis rationale

## Abbreviations

TFs: Transcription factors
CMA: Comprehensive Meta-Analysis
PEG: Polyethylene glycol
CIs: Confidence intervals
REL: Relative electrolyte leakage
SOD: Superoxide dismutase
TC: Transgenic plants
NC: Non-transgenic plants
CBF: C-repeat Binding Factor
DREB: Dehydration Responsive Element Binding
RVE 1: REVEILLE1
CIR: CIRCADIAN1
STO: Salt tolerance
SOS: Salt overly sensitive

## Additional Information and Declarations

### Data Availability

The following information was supplied regarding data availability: The raw measurements are available in the Supplemental Files.
