# Supplementary material for: Meta-analysis of the effect of expression of MYB transcription factor genes on abiotic stress
Source: PeerJ. 2021 Jun 8;9:e11268. doi: 10.7717/peerj.11268 (PMC8194419; doi:10.7717/peerj.11268)
Supplement: Supplemental Information 1 [file peerj-09-11268-s001.doc]

| **Section/topic** | **#** | **Checklist item** | **Reported on page #** |
| --- | --- | --- | --- |
| **TITLE** | | |  |
| Title | 1 | The report is identified as a meta-analysis. |  |
| **ABSTRACT** | | |  |
| Structured summary | 2 | The structured abstract includes Objective, Research Design and Methods, Results and Conclusion. |  |
| **INTRODUCTION** | | |  |
| Rationale | 3 | Described in the Introduction. | Page on 2-3 |
| Objectives | 4 | Stated in the Introduction. | Page on 2-3 |
| **METHODS** | | |  |
| Protocol and registration | 5 | The protocol is described in the Materials and methods. Registration does not apply. |  |
| Eligibility criteria | 6 | Stated in the Materials and methods. | page on 4 |
| Information sources | 7 | Stated in the Materials and methods. | page on 4 |
| Search | 8 | Stated in the Materials and methods. | page on 4 |
| Study selection | 9 | Stated in the Materials and methods. | page on 4-7 |
| Data collection process | 10 | Described in the Materials and methods. | page on 4-7 |
| Data items | 11 | Described in the Materials and methods. | page on 4-7 |
| Risk of bias in individual studies | 12 | Stated in the Materials and methods. | page on 5-7 |
| Summary measures | 13 | Stated in the Materials and methods. | page on 5 |
| Synthesis of results | 14 | Stated in the Materials and methods. | page on 6-7 |

Page 1 of 2

| **Section/topic** | **#** | **Checklist item** | **Reported on page #** |
| --- | --- | --- | --- |
| Risk of bias across studies | 15 | Stated in the Materials and methods. | page on 6-7 |
| Additional analyses | 16 | Described in the Materials and methods. | page on 6-7 |
| **RESULTS** | | |  |
| Study selection | 17 | Stated in the Results. | page on 7 |
| Study characteristics | 18 | Stated in the Results. | page on 7-10 |
| Risk of bias within studies | 19 | Stated in the Results. | page on 7 |
| Results of individual studies | 20 | Stated in the Results. | page on 7-10 |
| Synthesis of results | 21 | Stated in the Results. | page on 7-13 |
| Risk of bias across studies | 22 | Stated in the Results. | page on 7 |
| Additional analysis | 23 | Stated in the Results. | page on 10-13 |
| **DISCUSSION** | | |  |
| Summary of evidence | 24 | Stated in the Discussion. | page on 14-17 |
| Limitations | 25 | Stated in the Discussion. | page on 14-15 |
| Conclusions | 26 | Stated in the Discussion. | page on 17 |
| **FUNDING** | | |  |
| Funding | 27 | This research was supported by the National Natural Science Foundation of China (31972460, 31870680), the earmarked fund for China Agriculture Research System (CARS-19), Jiangsu Agricultural Industry Technology System (JATS[2019]423), Nanjing Science & Technology Project (2019RHJD203). We are thankful the above funds for providing financial support for execution of this research work.  The funder of these funding is Professor Wanping Fang, one of the authors of this article, who provided funding and provided revised suggestions.  Stated in the Funding. | page on 18 |

*From:*  Moher D, Liberati A, Tetzlaff J, Altman DG, The PRISMA Group (2009). Preferred Reporting Items for Systematic Reviews and Meta-Analyses: The PRISMA Statement. PLoS Med 6(7): e1000097. doi:10.1371/journal.pmed1000097

For more information, visit: **www.prisma-statement.org**.

Page 2 of 2
