# Supplementary material for: Meta-analysis of the effect of expression of MYB transcription factor genes on abiotic stress
Source: PeerJ. 2021 Jun 8;9:e11268. doi: 10.7717/peerj.11268 (PMC8194419; doi:10.7717/peerj.11268)
Supplement: Supplemental Information 6 [file peerj-09-11268-s006.docx]

Meta-analysis refers to the statistical synthesis of results from multiple studies (Borenstein et al., 2009). The analysis allows us to characterize the consistency of a treatment effect and to estimate its magnitude much more precisely than from single studies alone. It also enables us to quantify the influence of various experimental factors on the treatment effect. These factors that have been investigated across many studies and whose nuances go far beyond the capability of any one experiment.

MYB transcription factor gene is the most studied gene in plant stress response pathway. Since it was first discovered in early 1980’s, a large body of literature has been generated in terms of gene characterization from different plant species and have been over-expressed in the same or different species for stress tolerances, including drought, chilling or freeze, and salt responses. Although there have been quite a few descriptive, narrative reviews, but there have not been any systematic analysis in an unbiased way.

We performed a meta-analysis on overexpression of MYB genes to summarize their effects on plant responses to abiotic stress. Additionally, we examined several experimental factors (often termed moderator variables, or simply, moderators) that may have influenced the extent of the MYB effect on plant response. The analysis points to several potential research areas which should help scholars better understand the roles of the MYB gene family in improving the resistance

We sought to answer the following questions:

(i) What is the overall impact of MYB transformation across studies on plant response to stress?

(ii) Has the MYB influence been more pronounced in stressed plants than in unstressed plants?

(iii) How have particular experimental variables affected the size of MYB influence?

The first paper of our meta-analysis results and which is the first paper in this field, has been published in Plant Biotechnology Journal in 2017, and so far it has been cited 19 times (data from web of science). And we also published another results of meta-analysis in Environmental and Experimental Botany in 2017, and it has been cited 10 times (data from web of science). After 2017, we also published two paper of meta-analysis in Frontiers in Plant Science. Our results provide some new ideas and foundations for researchers in this field. The application of Meta-analysis in biotechnology field is relatively new, and we think it meets the scope of **Peer J**.

**Peer J** is an open access scholarly journal, and we hope to have more researchers in the same field to share our results. It would be a great honor for us to be considered by your journal.

[1] Ma Y C , Augé, Robert M, Dong C , et al. Increased salt tolerance with overexpression of cation/proton antiporter 1 genes: a meta‐analysis[J]. Plant Biotechnology Journal, 2017.

[2] Chao, Dong, Yuanchun, et al. Meta-analysis of the effect of overexpression of CBF/DREB family genes on drought stress response[J]. Environmental & Experimental Botany, 2017.

[3] Yuanchun M , Qunkang C , Zongming C , et al. Identification of Important Physiological Traits and Moderators That Are Associated with Improved Salt Tolerance in CBL and CIPK Overexpressors through a Meta-Analysis[J]. Frontiers in Plant ence, 2017, 8.

[4] Chao D , Yuanchun M , Dan Z , et al. Meta-Analysis of the Effect of Overexpression of Dehydration-Responsive Element Binding Family Genes on Temperature Stress Tolerance and Related Responses[J]. Frontiers in Plant Science, 2018, 9:713.
